# Cardiometabolic Morbidity and Mortality with Smoking Cessation, Review of Recommendations for People with Diabetes and Obesity

**DOI:** 10.1007/s11892-020-01352-6

**Published:** 2020-12-08

**Authors:** Katarina Kos

**Affiliations:** Institute of Biomedical and Clinical Sciences, University of Exeter Medical School, University of Exeter, Exeter, Devon UK

**Keywords:** Smoking cessation, Type 2 diabetes, Nicotine, Tobacco, Obesity, E-cigarettes, Vaping

## Abstract

**Purpose of Review:**

Obesity is closely linked with the pathogenesis of type 2 diabetes (T2DM) and cardiovascular disease (CVD), and whilst smoking cessation is associated with weight gain, there are concerns that this weight gain may offset the benefit of CVD risk reduction especially in those with considerable post-cessation weight gain. The aim of this narrative review is to evaluate recent evidence on smoking cessation and cardiometabolic outcomes and discuss limitations of current knowledge and studies.

**Recent Findings:**

Nicotine is a key player in modulating energy balance by influencing lipid storage in adipose tissue by affecting lipolysis, energy input by modulating appetite and energy output by increasing sympathetic drive and thermogenesis. It also increases insulin resistance and promotes abdominal obesity. The CVD risk and mortality associated with cigarette smoking potentiate the CVD risks in patients with diabetes. Evidence supports the benefit of quitting cigarette smoking regardless of any subsequent weight gain. Data suggests that the cardiometabolic risk is limited to the first few years and that cardiovascular health and mortality benefit of smoking cessation outweighs the harm related to weight gain. This weight gain can be limited by nicotine replacement of which e-cigarettes (vaping) are increasingly popular if it is not an alternative to cigarette smoking. However, long-term health data on e-cigarettes is needed prior to formal recommendation for its use in smoking cessation.

**Summary:**

The recommendation for cessation of cigarette smoking is justified for those at high risk of weight gain and diabetes. However, for most benefit, consideration should be given for personalized weight management to limit weight gain. Awareness of a ‘lean paradox’ by which lower weight is associated with increased CVD risk may help to improve motivation and insight into the bias of smoking, health and body composition otherwise known to epidemiologists as the ‘obesity paradox’.

## Introduction

Smoking is pro-atherogenic and accelerates the progression of macro and microvascular disease and is an independent risk factor for premature mortality in patients with diabetes [1]. People with diabetes are a high-risk group for cardiovascular disease (CVD) even without smoking, mainly attributed to the chronic effects of hyperglycaemia. The American Diabetes Association and the United Kingdom’s National Institute of Health and Care (NICE) recommendation for subjects with diabetes is cigarette smoking cessation [2, 3]. However, whilst some weight (re)gain is inevitable, the efficacy of abstinence is greatly compromised by the unwillingness of smokers to gain even modest amounts of weight with quitting [4]. Obesity itself is a major independent risk factor for type 2 diabetes (T2DM) and cardiovascular disease to the extent of an exponentially increase of T2DM risk with each unit of BMI [5, 6]. The increasing rates of obesity in the USA have partially been attributed to a decline in smoking popularity [7]. Cigarette smoking rates in the USA were 45% in 1965 and 15.5% in 2016, and 5–6% of current smokers use e-cigarettes [1, 8, 9]. Smoking has been associated with the bias explaining the ‘obesity paradox’ [10]. Smoking increases adipose tissue breakdown (lipolysis), induces insulin resistance and leads to weight loss. When not accounting for smokers, the general population with obesity may appear to have lower CVD mortality than lean subjects such as those who smoke.

The conundrum of post-cessation weight gain and health has stimulated research and discussions on the cardiovascular benefit of smoking cessation in those who gain weight in excess [11]. This review explores the evidence of the interplay of diabetes and CVD morbidity and mortality with smoking cessation in the context of obesity and explores any potential bias. It challenges the hypothesis that weight gain after quitting smoking worsens diabetes and cardiovascular risk disproportionally to the extent that health-care professionals should be cautious about recommending smoking cessation.

## Smoking, Smoking Cessation and Weight

Smokers lose subcutaneous body fat through increased fat tissue breakdown (lipolysis) and a reduction in adipose tissue synthesis (lipogenesis) [12]. The fat distribution however appears to change unfavourably with an increase of the abdominal fat depot. Abdominal obesity is a parameter of the metabolic syndrome used in cardiovascular risk assessment, albeit the abdominal circumference parameter was a later addition to the original syndrome description by Reaven who, when first describing it, linked the pathogenesis of cardiovascular disease with insulin resistance [13, 14]. The degree of change in the abdominal fat distribution appears to have an association with the amount of cigarettes smoked as shown in a cross-sectional population study of 6123 Caucasians with the observation that with increasing cigarette usage, the odds ratio (OR) for abdominal obesity in men increased to 1.28 (95% confidence interval (CI): 0.78–2.10) for moderate smokers and 1.94 (1.15–3.27) for heavy smokers (*P* = 0.03 for trend) and 1.07 (0.72–1.58) and 2.15 (1.26–3.64) in female moderate and heavy smokers, respectively (*P* < 0.01 for trend) [15].

Apart from carcinogenic tar, cigarettes contain nicotine, which suppresses appetite as confirmed in rat experiments by a decrease in their meal size and lengthening of intervals between meals [16]. This is mediated by nicotine affecting appetite regulating hormones (peptide YY, orexin, neuropeptide Y), activation of proopiomelanocortin (POMC) and changes in expression of various hypothalamic receptors [12]. Nicotine also affects adipose tissue metabolism directly by an increase in lipolysis and affects lipases which regulate deposition of free fatty acids into adipose tissue as well as the secretion of fat hormones such as adiponectin [12]. A resulting surplus of FFA leads to lipotoxicity and contributes to insulin resistance.

Furthermore, nicotine increases neurotransmitter release and stimulates the sympathetic drive through which it can activate thermogenesis of brown adipose tissue and which further stimulates weight loss. It acts through an upregulation of cholinergic nicotinic receptors (nACHRs) peripherally and centrally. These receptors are also expressed in adipose tissue and have been discussed as potential drug targets for the treatment of obesity [17]. Nicotine also stimulates dopaminergic neurons of the lateral hypothalamus which are part of the reward system and induces addiction [18].

With quitting smoking and without treatment or drugs to assist cessation, a meta-analyses of 62 studies reported an average weight gain of 4.67 kg (95% confidence interval: CI 3.96–5.38) 12 months after continuous abstinence [19]. Results from the same meta-analysis showed that the weight gain is noticeable from the first months of cessation and progressively gained in the first year of quitting [19], suggesting that the weight gain is a direct result of abstinence. Longitudinal studies relating to cardiometabolic risk typically consider the weight regain at 6 to 8 years post-cessation [20••, 21••], albeit about 60% of weight regain occurs in the first year [19, 22].

Greater weight gain is found in previously heavier smokers (high Fagerström Score for nicotine dependence) and those with higher BMI before quitting, in women and those below age of 55 [19, 23–25]. More prone to post-cessation weight gain are smokers of low socio-economic status. Weight gain also depends on geographical region (higher in North America than Asia) and is more frequent in former smokers who control negative emotions with temporary gratification [12, 26, 27] Frequent unsuccessful attempts to quit smoking could lead to weight cycling and higher weight regain after every episode [26]. Reports on the proportion of people who gain more than 10 kg range from 13 to 15% [19, 21••, 24]. A study using self-reported weight gain showed that of those classified as historic binge eaters gained on average 11.4 kg (SD = 11.2) in comparison to 5.0 kg (SD = 6.9) in non-binge eating obese subjects, suggesting that former smokers may have replaced binge eating with smoking as a coping strategy [28].

Twin studies refute a contribution of genetic factors in these weight changes [29]. The weight change with smoking and its cessation is mediated largely through the cumulative actions of nicotine (or its withdrawal) [12]. Nicotine withdrawal increases calorie intake in the range of 250–300 kcals per day [26]. As mentioned above, nicotine affects fat hormones and storage in adipose tissue [30], and its withdrawal slows the metabolic rate, resets appetite and promotes addictive behaviour [12].

## Smoking and Metabolic Risk/Diabetes

A large meta-analysis including 88 prospective cohort studies concluded that active smoking was associated with an increased risk of incident T2DM, with a pooled relative risk (RR) of 1.37 (95% CI: 1.3–1.4) compared with never smokers [31]. Similar increases were found in reviews and reports from European, Asian and Japanese populations for incident diabetes as well as prediabetes [32, 33]. Globally, about 12–19% of T2DM in men and 3–5% in women were estimated to be attributable to smoking [31, 34]. About 27 million diabetes cases worldwide were linked directly to active smoking [31]. A meta-analysis of 22 prospective observational studies in Japanese subjects showed that T2DM increased by 16% for each increment of 10 cigarettes smoked per day [34]. A systematic review and meta-analysis concluded that people with diabetes who smoke have a lower HbA1C and less favourable lipid profile [35].

From experimental studies, we learn that smoking cigarettes decreases insulin sensitivity such as in young healthy individuals, smokers and non-smokers and worsens insulin resistance in those with existing type 2 diabetes [36–38]. In vivo experiments in mice exposed to nicotine had reduced insulin sensitivity [39]. Nicotine-mediated activation of AMPKα2 kinase and the respective increase in lipolysis in conjunction with weight loss is thought to contribute to the increased insulin resistance of cigarette smoking [10, 39]. Whilst there may be several factors to explain dyslipididaemia in humans [40], a clinical study on 11 heavy smokers who used transdermal nicotine replacement for cessation showed that insulin sensitivity only improved after nicotine withdrawal irrespective of weight gain [41], suggesting nicotine as a key player in its pathogenesis.

When comparing smokers with never smokers among people with diabetes, the systematic review and meta-analysis of Pan et al. found that the pooled RR was 1.44 (CI: 1.34–1.54) for total CVD (16 studies), 1.51 (95% CI: 1.4–1.6) for coronary heart disease (21 studies), 1.49 (1.3–1.7) for cardiovascular mortality (13 studies with 37,550 participants) and 1.55 (CI: 1.46–1.64) for total mortality (48 studies with 1,132,700 participants) [42].

## Smoking Cessation, Diabetes and Weight Change

The risk of new-onset diabetes continues to increase in the first few years after smoking cessation compared to never smokers and then begins to decline, and the risk becomes comparable to those who never smoked. In the meta-analysis of Pan et al. which included 10 prospective studies (1,086,608 participants) comparing never smokers with quitters, the risk of diabetes was increased, with a RR = 1.54 (95% CI: 1.36–1.74) for those who had quit smoking less than 5 years before baseline, decreased progressively for those who stopped for 5–9 years to a RR of 1.18 and to a RR of 1.11 for those abstaining for ≥ 10 years [31]. This is similar to the findings from a meta-analysis of Japanese employees (*n* = 343,573) which showed a decline of diabetes risk reaching an RR of 1.00 (95% CI: 0.88–1.13) for long-term quitters (≥ 10 years) in comparison to never smokers; however, the influence of weight change was not explored [34]. This short-term elevated risk for T2DM is higher in the Asian population than in North Americans or Europeans [31].

The analysis of data from US populations including the Nurses’ Health Study (NHS), NHS II and the Health Professionals Follow-Up Study (HPFS) by Hu et al. on progression of diabetes in those without prevalent diabetes, CVD or any chronic disease at baseline [21••] found that the diabetes risk is in direct proportion to weight gain after smoking cessation (weight change within 6 years of cessation explained 68% of elevated diabetes risk in the mediation analysis). This risk was highest in the quitters who gained more than 10 kg (hazard ratio (HR) = 1.59 (95% CI: 1.36–1.85)), peaked at 5 to 7 years from quitting and was overall higher in former smokers than in current smokers (HR of 1.22, 1.12–1.32). The weight gain did not attenuate the apparent benefits of smoking cessation on reducing cardiovascular mortality or extending longevity (see Table 1). The diabetes risk was not increased in those without weight gain [21••].

**Table 1:** Key studies on smoking cessation and cardiometabolic risk with appreciation of weight change

Reference	Cohort	Primary outcome	Weight considerations	Findings
Smoking cessation and weight change in relation to cardiovascular disease incidence and mortality among patients with type 2 diabetes: a population-based cohort study (Liu G, 2020) [20••]	NHS and HPFS (173,229 participants of which 10,809 had diabetes, follow-up measured in person-years)	Incidence of CVD, CHD and mortality	Focus on weight gain 6 years after quitting	CVD: HRs were 0.83 (95% CI: − 0.70–0.99) among all recent quitters, 0.77 (0.62–0.95) among recent quitters without weight gain, 0.99 (0.70–1.41) among recent quitters with weight gain of 0.1 to 5.0 kg, 0.89 (0.65–1.23) among recent quitters with weight gain > 5.0 kg and 0.72 (0.61–0.84) among longer-term quitters Mortality: In comparison to smokers, HR 0.69 (0.58–0.82) among long-term quitters without weight gain within 6 years following cessation, 0.57 (0.45–0.71) among long-term quitters with weight gain of 0.1 to 5.0 kg and 0.51 (0.42–0.62) among long-term quitters with weight gain of more than 5.0 kg
Smoking cessation, weight gain and the trajectory of estimated risk of coronary heart disease: 8-year follow-up from a prospective cohort study (Chen et al., 2019) [43•]	Japanese cohort of middle-aged male employees (*n* = 18,562)	10-year CHD risk	Weight gain of < 5 kg and > 5 kg at 8-year follow-up (no stratification to diabetes)	CHD risk decreased more rapidly in quitters without weight gain than in quitters with weight gain with a change rate of − 0.90% per year (95% CI: − 1.04 to − 0.75) versus − 0.40%/year (− 0.60 to − 0.19)
Smoking cessation, weight change, type 2 diabetes and mortality (Hu et al., 2018) [21••]	Longitudinal cohort study with data from NHS, NHSII and HPFS with exclusion of people with prevalent T2DM (> 162, 000 participants followed up over 19.6 years from 1984)	New type 2 diabetes risk, CVD and all-cause mortality	Weight gain within 6 years	T2DM risk: HR of 1.08 (95% confidence interval CI: 0.93–1.26) among recent quitters without weight gain, 1.15 (0.99–1.33) among those with weight gain of 0.1 to 5.0 kg, 1.36 (1.16–1.58) among those with weight gain of 5.1 to 10.0 kg and 1.59 (1.36 1.85) among those with weight gain of more than 10.0 kg CVD risk: 0.69 (95% CI, 0.54–0.88) among recent quitters without weight gain, 0.47 (0.35–0.63) among those with weight gain of 0.1 to 5.0 kg, 0.25 (0.15–0.42) among those with weight gain of 5.1 to 10.0 kg, 0.33 (0.18–0.60) among those with weight gain of more than 10.0 kg and 0.50 (0.46–0.55) among longer-term quitters (> 6 years since smoking cessation)
Association of smoking cessation and weight change with cardiovascular disease among adults with and without diabetes (Claire et al., 2013) [44]	Framingham Offspring Study collected from 1984 through 2011 (*n* = 3251)	CVD incidence over 6 years	4 year weight gain in recent quitters (< 4 years) which was 3.6 kg with T2DM and 2.7 kg without T2DM	CVD risk in those without diabetes: recent quitters HR of 0.47 (95% CI: 0.23–0.94) and HR of 0.46 (0.34–0.63) in long-term quitters compared with smokers No change in associations of CVD risk after weight adjustments. Underpowered for diabetes subgroup analysis
Smoking cessation, weight change and coronary heart disease among postmenopausal women with and without diabetes (Luo et al., 2013) [45]	Women’s Health Initiative Study of 161,808 postmenopausal women recruited between 1993 and 1998	CHD risk	Weight gain between baseline and year 3 (categorized as < 5 kg, 5−< 10 kg or ≥ 10 kg)	Among 6338 women with diabetes, those who had newly quit had a lower risk for CHD with an HR of 0.36 (95% CI: 0.17–0.78), as did former smokers: HR of 0.41 (0.29–0.59) compared with current smokers. These associations were unchanged after further adjustment for weight change

When considering glycaemic control, a Chinese prospective study showed that the risk of poor glycaemic control was higher in smokers, and this risk normalized after at least 10 years of abstinence [46]. The same pattern was found for fasting glucose and HbA1C [46]. In comparison, studies of shorter duration did not find any difference in glycaemic control after stopping smoking for 1 year [47]. Evidence reviews report that quitting smoking reduces macrovascular diabetes complications and nephropathy; the evidence however is less conclusive in regard to other microvascular complications [48].

## Smoking Cessation and CVD in Relation to Weight Change

Cigarette smoking is independently associated with worsening atherosclerosis/hypertension, dyslipidaemia and increased cardiovascular morbidity and mortality. Smoking cessation reduces these risks [49]. The multiple effects of nicotine on adipose tissue itself play an important role in the pathogenesis of atherosclerosis [50].

Data from a prospective cohort study of a Finish population (*n* = 60,000) with and without T2DM reported a coronary heart disease (CHD) mortality risk sixfold higher for male smokers with T2DM with an HR = 6.15 (95% CI = 4.2–8.9) in comparison to HR of 2.62 (95% CI = 1.60–4.3) in non-smoking men with T2DM. Similarly for women with diabetes, the HRs (95% CIs) for CHD mortality were 6.92 (2.79–17.19) in smokers and 4.06 (2.83–5.82) in non-smokers [51].

Liu and colleagues examined the risk of CVD and mortality in relation to weight gain after smoking cessation in health-care professionals with T2DM from the Nurses’ Health Study (1976–2014) and the Health Professionals Follow-Up Study (1986–2014). Their analyses considered diabetes duration and the dynamics of the quitting process by inclusion of transient quitters [20••]. An attenuation of the benefit in the incident CVD risk in those with weight gain was found when compared to those without weight gain. Among recent quitters of 2–6 consecutive years, the multivariate-adjusted HR for CVD was 0.77 (95% CI: 0.62–0.95) among those without weight gain, 0.99 (95% CI: 0.7–1.4) among those with weight gain of 0.1–5.0 kg and 0·89 (95% CI: 0.65–1.2) among recent quitters with weight gain > 5·0 kg with the same pattern irrespective of diabetes status [20••]. No explanation was found for the lower benefit in the 0.1–0.5 kg weight gain group, and the influence of diabetes control was not taken into account. Similarly, attenuation of the benefit of smoking cessation was reported in a Japanese cohort of middle-aged male employees showing that the 10-year CHD risk reduction was greatest in the first year of cessation in those with < or > than 5 kg of weight gain with a change rate of − 0.90% (95% CI: − 1.04 to − 0.75) versus − 0.40% (− 0.60 to − 0.19) and that the benefit was sustained regardless of weight gain in the subsequent years [43•]. Albeit there was no differentiation of people with diabetes, the latter study showed that glucose levels increased in those with more weight gain [43•]. Clair et al. studied the Framingham Offspring Population (*n* = 3251of which 11.4% had diabetes at baseline) with data collected from 1984 to 2011 for the 6-year incidence of CVD events in smokers and quitters who lost or gained weight. Due to the small numbers of events, the subgroup analyses considering weight gain was limited but suggested a CVD risk benefit in diabetic participants regardless of weight gain qualitatively similar to non-diabetic subjects; however, this did not reach statistical significance, possibly due to limited study power [44]. A study in postmenopausal women in the Women’s Health Initiative study (WHI, *n* = 161,808; see Table 1) was similarly not able to make conclusions in the diabetes subgroups (5–7% of the population had diabetes) but showed improvement in CHD with smoking cessation in people with no diabetes regardless of weight gain [45].

A substantial improvement was also reported in all-cause mortality regardless of post-cessation weight. Liu et al. report all-cause mortality among long-term quitters > 6 years since smoking cessation with a multivariate-adjusted HR of 0.69 (95% CI: 0.58–0.82) among those without weight gain, 0.57 (95% CI: 0.45–0.71) among those with weight gain of 0.1–5.0 kg and 0.51(95% CI: 0.42–0.62) among those with weight gain of > 5·0 kg [20••]. Key studies considering post-cessation weight gain are shown in Table 1.

## E-cigarette Smoking

E-cigarettes are gaining popularity with 60% of smokers in the USA using e-cigarettes at least on some days if not regularly [52, 53]. A UK survey found that about 6% of people used e-cigarettes in 2017, most of them (52%) were previous smokers [54]. E-cigarettes also known as electronic nicotine delivery systems (ENDS) are battery-driven devices that vaporize the nicotine present in a refill liquid. Vaping has become the most popular method for smoking cessation [9]. A worldwide online survey in 10 languages showed that many people use e-cigarettes in combination to cigarette smoking as dual use [55]. The same survey found that only 3.5% of smokers use non-nicotine containing vapes [55].

Nicotine replacement has been used since the 1980s commonly as dermal patches, inhalers, gum, etc. and found effective for improving the symptoms of smoking cessation [12]. This has since been replicated with the use of nicotine containing e-cigarettes which ameliorate post-cessation weight gain [56] and maybe more effective than traditional nicotine replacement therapies in quitting success as shown in smokers attending UK NHS smoking services who achieved a 1-year abstinence of 18% when nicotine e-cigarettes were combined with behaviour support [57]. A New Zealand survey showed that nicotine e-cigarettes lead to abstinence in more people (7.3%) at 6 months than placebo e-cigarettes (risk difference 3.16 (95% CI: − 2·29 to 8.61) [58]. Cohort studies however report only a modest quit rate which is no different in dual users [59, 60]. A recent meta-analyses [61] concluded that there is only very limited evidence regarding their impact on smoking cessation [54]. This is in agreement with a recent survey in England, which shows that E-cigarette use is associated with only a modest increased quit rate [62].

Recent surveys show that many quitters are not aware of potential weight benefits with use of nicotine e-cigarettes [53, 63]. The quit rate especially in people with diabetes is low, and whether the awareness of weight benefit could improve quit rates remains to be shown. A survey of English smokers concluded that one in eight people would stop smoking if they were convinced that vaping prevented gain weight [63]. Several studies on vaping in regards its weight benefit are ongoing, and some researchers discuss nicotine vaping as intervention for the management of obesity per se [63–65].

Challenges for the assessment of e-cigarettes’ efficiency and metabolic effects are the appreciation of nicotine dosage. Their content varies, and regulations differ among countries, e.g. a Juul pod in the USA can contain up to 59 mg/mL of nicotine compared with 18 mg/mL in the UK, whilst a cigarette contains average of 10–12 mg, of which only 1% is estimated to be inhaled [66]. In addition, it is difficult to estimate the rate of absorbance of nicotine as this depends on the vaping technique, and modern devices appear to allow a larger absorbance [55]. Such a direct comparison of any vaping ‘unit’ with corresponding number of cigarettes for equal absorption of nicotine is not possible.

Vaping carries health risks as demonstrated by increasing number of reports of acute lung injury (> 1900 in the USA). These present as infiltrates on chest imaging and are attributed to various toxic compounds and ingredients [67–69]. The exact pathophysiology of acute lung inflammation is yet unclear nor are long-term health effects of vaping [70]. This is particularly important for those choosing vaping for the long term rather than for smoking cessation per se and increasing number of people not using e-cigarettes for this purpose [20••, 21••]. As outlined above, whilst nicotine impairs glucose tolerance and influences the vascular system with an increased sympathetic activation, concerns have been raised regarding worsening glucose impairment and cardiovascular risk with nicotine replacements [41, 71]. Reports on mice and humans so far do not detect changes in insulin resistance with e-cigarette use [72]; however, this may be nicotine dose dependent, and smokers using nicotine containing e-cigarettes may expose themselves with lower doses than with tobacco containing classical cigarettes. E-cigarettes are not approved by the FDA at time of writing this review, and the NICE review in 2018 advises the need of more evidence on long-term safety prior to a possible recommendation [73].

## Evidence Limitations

Limitations of studies on smoking cessation and cardiometabolic risk include fairly small sample sizes, especially in subgroups of high weight gain and diabetes. Some studies use point prevalence measures of non-smoking status rather than capturing dynamic changes in smoking status. Even those who manage abstinence for 1 year, about 30% have been reported to relapse in the following decade [74]. Conditions which affect weight change and lifestyle behaviour need to be excluded to avoid bias by reverse causality such as the ‘sick quitter effect’ [11]. Abrupt smoking cessation and complete abstinence may be more typical of a person with a new diagnosis of a health condition.

Many studies are based on self-reporting; this can include smoking behaviour; however, using objective measure of smoking, e.g. cotinine levels, is preferable. Data on weight changes and often the presence and duration of diabetes are also frequently obtained by self-reporting. The diabetes control at time of smoking cessation should be taken into account as this will influence future weight.

**Fig. 1:**
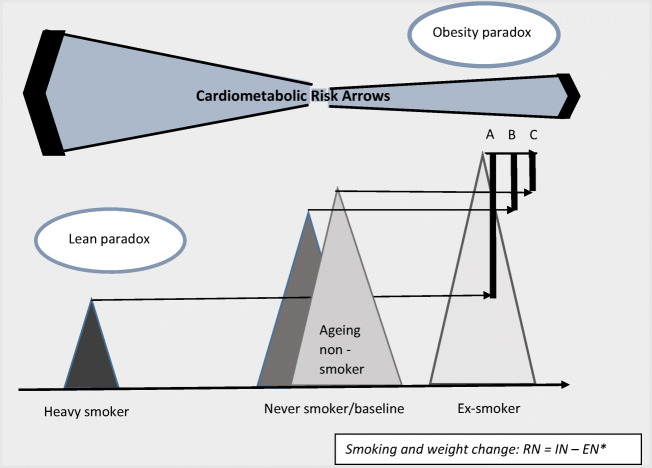
Weight and cardiometabolic risk changes in relation to the smoking severity and status. Figure demonstrating weight changes as a function of smoking intensity and status. An obese smoker weighs less with heavy cigarette smoking, and the leaner smoking state is associated with higher CVD morbidity and mortality than the obese state (lean paradox = the lean habitus does not appear to offer protection from CVD otherwise found with lower BMI). There is a natural weight gain with ageing which should be taken into account when referring to quitting induced weight gain. The weight differences with smoking cessation are notified by: A which refers to the weight gain with smoking cessation which is not taking account the weight difference from the pre-smoking weight shown with B. C takes account for the age-related weight gain. *Energy balance equation adjusted to smoking in which R is the rate of kcal/d that is stored or lost (and thus defines weight change), I is the intake of kcal/d, E is the rate of kcal/d expended and N is the nicotine dose (which does not reach 0 as nicotine is a natural ingredient of vegetables)

With nicotine being a key player in modulating weight change, we propose that the dosage of nicotine should be taken into account when evaluating efficacy and risks. Using the basic energy balance formulation used in the nutrition and physiology literature and adjusting it to nicotine (see Fig. 1) [75], its independent effects on all variables of energy balance become apparent: energy intake (by reducing appetite), energy expenditure (e.g. by increasing sympathetic drive) and energy storage (by affecting adipose tissue’s fat deposition enzymes). Mathematical modelling studies of changes in energy balance and respective weight change need to accept the physiological impact of nicotine on energy metabolism.

Use of smoking cessation strategies even if they have been used only intermittently or combined needs to be appreciated when referring to weight or glucose metabolism studies. Whilst no increased CVD risk has been observed with the use of drugs assisting smoking cessation such as bupropion and varenicline [33], they may affect post-cessation weight gain.

## Conclusion and Future Perspective

Stopping cigarette smoking reduces cardiovascular risk and mortality even in those with weight gain as result of smoking cessation, albeit ameliorating the benefit when considerate weight is gained. Smoking cessation thus benefits people with and without diabetes. As shown in Fig. 1, in order to estimate the cardiometabolic benefit, the ill effects of smoking need to be taking into account. As regards post-cessation weight gain, weight changes should to be related to the pre-smoking baseline weight corrected for weight gain with ageing once quitting commences. According to epidemiological studies, the weight gain through the middle adult age is estimated to be 0.5 to 1 kg per year [76]. The obesity paradox by which obesity is favourable over the lean smoking state holds true with smoking and CVD risk. Epidemiological studies have to correct for smoking as a strong statistical confounder to avoid bias in the relationship of BMI and cardiometabolic health [10]. In doing so, the assumption of an ‘obesity paradox’ can also be removed in ageing populations [77]. The considerable effect of nicotine on energy balance and appetite, by which it influences body weight, and the risk of diabetes, especially in heavy smokers (> 25 cigarettes a day), becomes apparent by this paradoxical BMI and CVD risk correlation [10, 23]. To the individual smoker, however, the term ‘lean paradox’ might be easier to understand and aid to the acceptance of post quitting weight gain by explaining that the lower smokers’ body weight is unfavourable and as the evidence presented suggests carries a higher CVD risk. Patients can be reassured that whilst some weight gain with smoking cessation is physiological, it is modifiable and the ‘lesser evil’ in comparison to cigarette smoking.

A proactive approach is needed with appropriate personalized cessation strategies to limit weight regain, especially for those at particular risk such as for heavy smokers with obesity and diabetes, and subjects who require emotional control are prone to binge eating. Studies are underway to trial scalable psychological interventions such as cognitive behaviour therapies and mindfulness-based therapies [78]. Whilst initial findings do not support a worsening in insulin resistance, little is yet known about the cardiometabolic and health risks of long-term e-cigarette use [73].

Emerging evidence from a meta-analysis shows that smoking depresses pulmonary immune function and increases progression of the COVID-19 infection with an OR of 1.91 (CI: 1.42–2.59) [79]. As this appears to be mediated by the nicotinergic nACHR7 receptor, it cannot be excluded that the use of nicotine-based products in general increases this risk [80]. Time will show whether the COVID-19 pandemic will motivate to healthier lifestyles and for quitting.

## References

[1] Haire-Joshu D, Glasgow RE, Tibbs TL (1999). Smoking and diabetes. Diabetes Care.

[2] Overview | Smoking: harm reduction | Guidance | NICE. https://www.nice.org.uk/guidance/ph45. Accessed 17 Nov 2020.

[3] Association, A. D. Lifestyle management: standards of medical care in Diabetes. Diabetes Care. 2018;41:S38–S50. 10.2337/dc18-S004 29222375

[4] Levine MD, Bush T, Magnusson B, Cheng Y, Chen X. Smoking-related weight concerns and obesity: differences among normal weight, overweight, and obese smokers using a telephone tobacco quitline. Nicotine Tob Res. 2013;15:1136–40. 10.1093/ntr/nts226 PMC3693501 23100456

[5] Haslam DW, James WPT. Obesity. Lancet. 2005;366:1197–209. 10.1016/S0140-6736(05)67483-1 16198769

[6] Colditz GA (1990). Weight as a risk factor for clinical diabetes in women. Am J Epidemiol.

[7] Flegal KM, Troiano RP, Pamuk ER, Kuczmarski RJ, Campbell SM (1995). The influence of smoking cessation on the prevalence of overweight in the United States. N Engl J Med.

[8] Jamal A, Phillips E, Gentzke AS, Homa DM, Babb SD, King BA, et al. Current cigarette smoking among adults — United States, 2016. Morb Mortal Wkly Rep. 2018;67:53–9. 10.15585/mmwr.mm6702a1 PMC5772802 29346338

[9] Caraballo RS, Shafer PR, Patel D, Davis KC, McAfee TA. Quit methods used by us adult cigarette smokers, 2014-2016. Prev Chronic Dis. 2017;14:160600. 10.5888/pcd14.160600. 10.5888/pcd14.160600 PMC5392446 28409740

[10] Yu XY, Song P, Zou MH (2018). Obesity paradox and smoking gun a mystery of statistical confounding?. Circ Res.

[11] Kos K (2020). Smoking cessation, weight gain, and cardiovascular risk. Lancet Diabetes Endocrinol.

[12] Filozof C, Fernández Pinilla MC, Fernández-Cruz A (2004). Smoking cessation and weight gain. Obes Rev.

[13] Reaven GM (1988). Role of insulin resistance in human disease. Diabetes.

[14] Eckel RH, Grundy SM, Zimmet PZ. The metabolic syndrome. Lancet. 2005;365:1415–28. 10.1016/S0140-6736(05)66378-7 15836891

[15] Clair C, Chiolero A, Faeh D, Cornuz J, Marques-Vidal P, Paccaud F, et al. Dose-dependent positive association between cigarette smoking, abdominal obesity and body fat: cross-sectional data from a population-based survey. BMC Public Health. 2011;11:23. 10.1186/1471-2458-11-23 PMC3025841 21223575

[16] Miyata G, Meguid MM, Varma M, Fetissov SO, Kim HJ (2001). Nicotine alters the usual reciprocity between meal size and meal number in female rat. Physiol Behav.

[17] Mcfadden KL, Cornier M-A, Tregellas JR, Picciotto M, Sproesser G. The role of alpha-7 nicotinic receptors in food intake behaviors. 2014;5:553. 10.3389/fpsyg.2014.00553. 10.3389/fpsyg.2014.00553 PMC4047526 24936193

[18] Li MD, Parker SL, Kane JK (2000). Regulation of feeding-associated peptides and receptors by nicotine. Mol Neurobiol.

[19] Aubin HJ, Farley A, Lycett D, Lahmek P, Aveyard P. Weight gain in smokers after quitting cigarettes: Meta-analysis. BMJ. 2012;345. 10.1136/bmj.e4439. 10.1136/bmj.e4439 PMC3393785 22782848

[20] •• Liu G, et al. Smoking cessation and weight change in relation to cardiovascular disease incidence and mortality in people with type 2 diabetes: a population-based cohort study. Lancet Diabetes Endocrinol. 2020. 10.1016/S2213-8587(19)30413-9. **Most recent paper with one of the largest population of people with Type 2 diabetes with appreciation of important cofounders.** 10.1016/S2213-8587(19)30413-9 PMC6986932 31924561

[21] •• Hu Y, et al. Smoking cessation, weight change, type 2 diabetes, and mortality. N Engl J Med. 2018;379:623–32. **Large study with appreciation of new onset of diabete 2 diabetes and smoking associated weight change over a long follow up period of 19.4 years.** 10.1056/NEJMoa1803626 PMC6165582 30110591

[22] O’Hara P (1998). Early and late weight gain following smoking cessation in the lung health study. Am J Epidemiol.

[23] Veldheer S, Yingst J, Zhu J, Foulds J (2015). Ten-year weight gain in smokers who quit, smokers who continued smoking and never smokers in the United States, NHANES 2003-2012. Int J Obes.

[24] Lycett D, Munafò M, Johnstone E, Murphy M, Aveyard P (2011). Associations between weight change over 8 years and baseline body mass index in a cohort of continuing and quitting smokers. Addiction.

[25] Komiyama M (2013). Analysis of factors that determine weight gain during smoking cessation therapy. PLoS One.

[26] Chiolero A, Faeh D, Paccaud F, Cornuz J. Consequences of smoking for body weight, body fat distribution, and insulin resistance 1,2. 2008. https://academic.oup.com/ajcn/article-abstract/87/4/801/4633357. 10.1093/ajcn/87.4.801 18400700

[27] Tian J, Venn A, Otahal P, Gall S (2015). The association between quitting smoking and weight gain: a systemic review and meta-analysis of prospective cohort studies. Obes Rev.

[28] White MA, Masheb RM, Grilo CM (2010). Self-reported weight gain following smoking cessation: a function of binge eating behavior. Int J Eat Disord.

[29] Piirtola M, Jelenkovic A, Latvala A, Sund R, Honda C, Inui F, et al. Association of current and former smoking with body mass index: a study of smoking discordant twin pairs from 21 twin cohorts. PLoS One. 2018;13:e0200140. 10.1371/journal.pone.0200140 PMC6042712 30001359

[30] Ferrara CM, Kumar M, Nicklas B, McCrone S, Goldberg AP (2001). Weight gain and adipose tissue metabolism after smoking cessation in women. Int J Obes.

[31] Pan A, Wang Y, Talaei M, Hu FB, Wu T (2015). Relation of active, passive, and quitting smoking with incident type 2 diabetes: a systematic review and meta-analysis. Lancet Diabetes Endocrinol.

[32] Aeschbachera S, et al. Association of smoking and nicotine dependence with pre-diabetes in young and healthy adults. Swiss Med Wkly. 2014;144:w14019. 10.4414/smw.2014.14019. 10.4414/smw.2014.14019 25295968

[33] Campagna D, et al. Smoking and diabetes: Dangerous liaisons and confusing relationships. Diabetol Metabolic Syndrome, 11. 2019;11:35. 10.1186/s13098-019-0482-2. 10.1186/s13098-019-0482-2 PMC6813988 31666811

[34] Akter S, Goto A, Mizoue T (2017). Smoking and the risk of type 2 diabetes in Japan: a systematic review and meta-analysis. J Epidemiol.

[35] Kar D, et al. Relationship of cardiometabolic parameters in non-smokers, current smokers, and quitters in diabetes: a systematic review and meta-analysis. Cardiovasc Diabetol. 2016;15, 158. 10.1186/s12933-016-0475-5. 10.1186/s12933-016-0475-5 PMC5121966 27881170

[36] Attvall S, Fowelin J, lager I, Von Schenck H, Smith U (1993). Smoking induces insulin resistance—a potential link with the insulin resistance syndrome. J Intern Med.

[37] Frati AC, Iniestra F, Ariza CR (1996). Acute effect of cigarette smoking on glucose tolerance and other cardiovascular risk factors. Diabetes Care.

[38] Targher G, Alberiche M, Zenere MB, Bonadonna RC, Muggeo M, Bonora E (1997). Cigarette smoking and insulin resistance in patients with noninsulin-dependent diabetes mellitus 1. J Clin Endocrinol Metab.

[39] Wu Y, Song P, Zhang W, Liu J, Dai X, Liu Z, et al. Activation of AMPKα2 in adipocytes is essential for nicotine-induced insulin resistance in vivo. Nat Med. 2015;21:373–82. 10.1038/nm.3826 PMC4390501 25799226

[40] Jensen EX, Fusch CH, Jaeger P, Peheim E, Horber FF (1995). Impact of chronic cigarette smoking on body composition and fuel metabolism. J Clin Endocrinol Metab.

[41] Assali AR, Beigel Y, Schreibman R, Shafer Z, Fainaru M (1999). Weight gain and insulin resistance during nicotine replacement therapy. Clin Cardiol.

[42] Pan A, Wang Y, Talaei M, Hu FB (2015). Relation of smoking with total mortality and cardiovascular events among patients with diabetes mellitus: a meta-analysis and systematic review. Circulation.

[43] • Chen S, et al. Smoking cessation, weight gain, and the trajectory of estimated risk of coronary heart disease: 8-year follow-up from a prospective cohort study. Nicotine Tob Res. 2019. 10.1093/ntr/ntz165. **Prospective study on CVD risk in Japanese male individuals stratefied by weight change.** 10.1093/ntr/ntz165 PMC7789946 31504860

[44] Clair C, Rigotti NA, Porneala B, Fox CS, D’Agostino RB, Pencina MJ, et al. Association of smoking cessation and weight change with cardiovascular disease among adults with and without diabetes. JAMA - J Am Med Assoc. 2013;309:1014–21. 10.1001/jama.2013.1644 PMC3791107 23483176

[45] Luo J, Rossouw J, Margolis KL (2013). Smoking cessation, weight change, and coronary heart disease among postmenopausal women with and without diabetes. JAMA - J Am Med Assoc.

[46] Peng K, Chen G, Liu C, Mu Y, Ye Z, Shi L, et al. Association between smoking and glycemic control in diabetic patients: results from the risk evaluation of cancers in Chinese diabetic individuals: a longitudinal (REACTION) study. J Diabetes. 2018;10:408–18. 10.1111/1753-0407.12625 29144059

[47] Iino K, Iwase M, Tsutsu N, Iida M (2004). Smoking cessation and glycaemic control in type 2 diabetic patients. Diabetes Obes Metab.

[48] Campagna D, Alamo A, di Pino A, Russo C, Calogero AE, Purrello F, et al. Smoking and diabetes: dangerous liaisons and confusing relationships. Diabetol Metab Syndr. 2019;11:85. 10.1186/s13098-019-0482-2 PMC6813988 31666811

[49] Perry IJ, Wannamethee SG, Walker MK, Thomson AG, Whincup PH, Shaper AG (1995). Prospective study of risk factors for development of non-insulin dependent diabetes in middle aged British men. BMJ.

[50] Wang Z, Wang D, Wang Y. Review Article Cigarette smoking and adipose tissue: the emerging role in progression of atherosclerosis. 2017:1–11. 10.1155/2017/3102737. 10.1155/2017/3102737 PMC5763059 29445255

[51] Barengo NC, Teuschl Y, Moltchanov V, Laatikainen T, Jousilahti P, Tuomilehto J (2017). Coronary heart disease incidence and mortality, and all-cause mortality among diabetic and non-diabetic people according to their smoking behavior in Finland. Tob Induc Dis.

[52] Reitsma MB, Fullman N, Ng M, Salama JS, Abajobir A, Abate KH, et al. Smoking prevalence and attributable disease burden in 195 countries and territories, 1990-2015: a systematic analysis from the global burden of disease study 2015. Lancet. 2017;389:1885–906. 10.1016/S0140-6736(17)30819-X PMC5439023 28390697

[53] Rutten LJ, et al. Use of e-cigarettes among current smokers: associations among reasons for use, quit intentions, and current tobacco use. Nicotine Tob Res. 2015 17:1228-34. 10.1093/ntr/ntv003. 10.1093/ntr/ntv003 PMC4592339 25589678

[54] El Dib R, et al. Electronic nicotine delivery systems and/or electronic non-nicotine delivery systems for tobacco smoking cessation or reduction: a systematic review and meta-analysis. BMJ Open. 2017;7. 10.1136/bmjopen-2016-01. 10.1136/bmjopen-2016-012680 PMC5337697 28235965

[55] Farsalinos KE, Romagna G, Tsiapras D, Kyrzopoulos S, Voudris V (2014). Characteristics, perceived side effects and benefits of electronic cigarette use: a worldwide survey of more than 19,000 consumers. Int J Environ Res Public Health.

[56] Russo C, Cibella F, Mondati E, Caponnetto P, Frazzetto E, Caruso M, et al. Lack of substantial post-cessation weight increase in electronic cigarettes users. Int J Environ Res Public Health. 2018;15:581. 10.3390/ijerph15040581. 10.3390/ijerph15040581 PMC5923623 29570695

[57] Hajek P, Phillips-Waller A, Przulj D, Pesola F, Myers Smith K, Bisal N, et al. A randomized trial of e-cigarettes versus nicotine-replacement therapy. N Engl J Med. 2019;380:629–37. 10.1056/NEJMoa1808779 30699054

[58] Bullen C, Howe C, Laugesen M, McRobbie H, Parag V, Williman J, et al. Electronic cigarettes for smoking cessation: a randomised controlled trial. Lancet. 2013;382:1629–37. 10.1016/S0140-6736(13)61842-5 24029165

[59] Jackson SE, Shahab L, West R, Brown J (2020). Associations between dual use of e-cigarettes and smoking cessation: a prospective study of smokers in England. Addict Behav.

[60] Choi K, Chen-Sankey JC. Will electronic nicotine delivery system (ENDS) use reduce smoking disparities? Prevalence of daily ENDS use among cigarette smokers. Prev Med Rep. 2020;17(101020). 10.1016/j.pmedr.2019.101020. 10.1016/j.pmedr.2019.101020 PMC6909091 31871881

[61] Kalkhoran S, Glantz SA (2016). E-cigarettes and smoking cessation in real-world and clinical settings: a systematic review and meta-analysis. Lancet Respir Med.

[62] Beard E, West R, Michie S, Brown J (2020). Association of prevalence of electronic cigarette use with smoking cessation and cigarette consumption in England: a time–series analysis between 2006 and 2017. Addiction.

[63] Jackson SE, Brown J, Aveyard P, Dobbie F, Uny I, West R, et al. Vaping for weight control: a cross-sectional population study in England. Addict Behav. 2019;95:211–9. 10.1016/j.addbeh.2019.04.007 PMC6555398 30981033

[64] Singh H, Kennedy RD, Lagasse LP, Czaplicki LM, Cohen JE (2018). E-cigarettes and weight loss-product design innovation insights from industry patents. Nicotine Tob Res.

[65] Glover M, Breier BH, Bauld L. Could vaping be a new weapon in the battle of the bulge? Nicotine Tob Res. 2017; 19:1536–1540. 10.1093/ntr/ntw278. 10.1093/ntr/ntw278 27798086

[66] Gotts JE, Jordt SE, McConnell R, Tarran R. What are the respiratory effects of e-cigarettes? BMJ. 2019;366. 10.1136/bmj.l5275 10.1136/bmj.l5275 PMC7850161 31570493

[67] Blagev DP, Harris D, Dunn AC, Guidry DW, Grissom CK, Lanspa MJ (2019). Clinical presentation, treatment, and short-term outcomes of lung injury associated with e-cigarettes or vaping: a prospective observational cohort study. Lancet.

[68] Rhoades DA, Comiford AL, Dvorak JD, Ding K, Hopkins M, Spicer P, et al. Perceptions of smoking and vaping on weight control among adult American Indians who smoke. J Community Health. 2019;44:1120–6. 10.1007/s10900-019-00694-x PMC6800397 31273619

[69] Layden JE, Ghinai I, Pray I, Kimball A, Layer M, Tenforde MW, et al. Pulmonary illness related to e-cigarette use in Illinois and Wisconsin - final report. N Engl J Med. 2020;382:903–16. 10.1056/NEJMoa1911614 31491072

[70] Christiani DC (2020). Vaping-induced acute lung injury. N Engl J Med.

[71] Eliasson B, Taskinen MR, Smith U (1996). Long-term use of nicotine gum is associated with hyperinsulinemia and insulin resistance. Circulation.

[72] Orimoloye OA, Uddin SMI, Chen LC, Osei AD, Mirbolouk M, Malovichko MV, et al. Electronic cigarettes and insulin resistance in animals and humans: results of a controlled animal study and the National Health and nutrition examination survey (NHANES 2013-2016). PLoS One. 2019;14:1228–35. 10.1093/ntr/ntq175. 10.1371/journal.pone.0226744 PMC6938328 31891598

[73] National Institute for Health and Care Excellence Smoking cessation interventions and services [C] Evidence reviews for advice on e-cigarettes on general sale NICE guideline NG92. (2018).

[74] Hawkins J, Hollingworth W, Campbell R. Long-term smoking relapse: a study using the British household panel survey. Nicotine Tob Res. 2010;12. 10.1093/ntr/ntq175 21036960

[75] Thomas DM, Ciesla A, Levine JA, Stevens JG, Martin CK (2009). A mathematical model of weight change with adaptation. Math Biosci Eng.

[76] Rockville MD. Agency for Healthcare Research and Quality US. 2013. 10.1080/15360280802537332 21923316

[77] Bowman K, Delgado J, Henley WE, Masoli JA, Kos K, Brayne C, et al. Obesity in older people with and without conditions associated with weight loss: follow-up of 955,000 primary care patients. J Gerontol A Biol Sci Med Sci. 2017;72:203–9. 10.1093/gerona/glw147 PMC5233914 27492450

[78] Vinci C. Cognitive behavioral and mindfulness-based interventions for smoking cessation: a review of the recent literature. Curr Oncol Rep. 2020;22:52. 10.1007/s11912-020-00915-w. 10.1007/s11912-020-00915-w PMC7874528 32415381

[79] Patanavanich R, Glantz SA. Smoking is associated with covid-19 progression: a meta-analysis. Nicotine Tob Res. 2020;22:1653–1656. 10.1093/NTR/NTAA082. 10.1093/ntr/ntaa082 PMC7239135 32399563

[80] Wang Q, Sundar IK, Li D, Lucas JH, Muthumalage T, McDonough SR, et al. E-cigarette-induced pulmonary inflammation and dysregulated repair are mediated by nAChR α7 receptor: role of nAChR α7 in SARS-CoV-2 Covid-19 ACE2 receptor regulation. Respir Res. 2020;21:154. 10.1186/s12931-020-01396-y PMC7301079 32552811

